# Preparation and Characterization of Bioactive Chitosan Film Loaded with Cashew (*Anacardium occidentale*) Leaf Extract

**DOI:** 10.3390/polym14030540

**Published:** 2022-01-28

**Authors:** Khursheed Ahmad Shiekh, Mooksupang Liangpanth, Siriporn Luesuwan, Rinlanee Kraisitthisirintr, Kittaporn Ngiwngam, Saroat Rawdkuen, Pornchai Rachtanapun, Thomas Karbowiak, Wirongrong Tongdeesoontorn

**Affiliations:** 1 School of Agro-Industry, Mae Fah Luang University, 333 Moo 1 Tasud, Chiang Rai 57100, Thailand; khursheed.research@mfu.ac.th (K.A.S.); 5951407003@lamduan.mfu.ac.th (M.L.); siriporn.lue@mfu.ac.th (S.L.); 6031407009@lamduan.mfu.ac.th (R.K.); 6251407001@lamduan.mfu.ac.th (K.N.); saroat@mfu.ac.th (S.R.); 2 Research Group of Innovative Food Packaging and Biomaterials Unit, Mae Fah Luang University, 333 Moo 1 Tasud, Chiang Rai 57100, Thailand; 3 Scientific and Technological Instruments Center, Mae Fah Luang University, 333 Moo 1 Tasud, Chiang Rai 57100, Thailand; 4 Division of Packaging Technology, School of Agro-Industry, Faculty of Agro-Industry, Chiang Mai University, Chiang Mai 50100, Thailand; pornchai.r@cmu.ac.th; 5 The Cluster of Agro Bio-Circular-Green Industry (Agro BCG), Chiang Mai University, Chiang Mai 50100, Thailand; 6 Center of Excellence in Materials Science and Technology, Chiang Mai University, Chiang Mai 50200, Thailand; 7 UMR PAM-Food and Wine Science and Technology, Agro-Sup Dijon, Université de Bourgogne France-Comte, Esplanade Erasme, F-21000 Dijon, France; thomas.karbowiak@agrosupdijon.fr

**Keywords:** chitosan, phytochemical extract, gas barrier, *Aspergillus niger*, antifungal film, eco-friendly, biopolymer film

## Abstract

Chitosan is a biopolymer known for its rapid biodegradability and film-forming properties. This research aimed to synthesize and characterize chitosan films loaded with cashew leaf extract (CLE) obtained from immature and mature cashew leaves via aqueous and 70% ethanolic extraction methods. Freeze-dried CLE samples were dissolved in 50% dimethyl sulfoxide for in vitro analysis and chitosan film preparation. The total phenolic content of mature cashew leaves extracted in ethanol (MECLE) showed higher free radicle scavenging activity by a 2,2-diphenyl-1-picrylhydrazyl assay than the other extracts (*p* < 0.05). MECLE displayed a lower minimal inhibitory concentration, minimum fungal concentration, and higher zone of inhibition against *Aspergillus niger* compared to the other treatments (*p* < 0.05). Film-forming solutions were prepared using 2% chitosan, 2% chitosan with 5% mature cashew leaves extracted in deionized water (MACLE) (*w*/*v*), and 2% chitosan with 5% MECLE (*w*/*v*), respectively, to cast films. Of these, 2% chitosan (CH) with 5% MECLE (CH-MECLE-5) displayed the highest thickness and water vapor transmission rate, water vapor permeability, and oxygen transmission rate when compared to other film samples (*p* < 0.05). The CH-MECLE-5 film showed the highest inhibition zone of *A. niger* compared to the control and treated films (*p* < 0.05). The lightness (L*) of the CH-MECLE-5 film decreased with increment in b* values, which represented the yellow color of the film. In addition, two-photon microscopy revealed a uniform distribution via the auto-fluorescent 3D structure of MECLE in the CH-MECLE-5 film. Therefore, chitosan combined with 5% MECLE may be a potential bioactive and eco-friendly packaging film.

## 1. Introduction

The application of synthetic and non-biodegradable polymer-tailored packaging films in foods has led to alarming consequences for the environment [1]. Excessive production of synthetic packaging materials may directly have an impact on the sustainability of non-renewable petroleum-based resources [2]. To neutralize the environmental constraints, alternative packaging films from biopolymers and agricultural residues have been proposed to achieve the goals of a sustainable green economy worldwide [3]. Biopolymers are most frequently exploited to develop biodegradable films that exhibit excellent bioactive and barrier properties compared to synthetic films [4]. Biopolymers can be obtained from the exoskeleton of crustaceans (chitosan), plants (cellulose, inulin, and starch), microbes (dextran and xanthan), and algae (alginate) [5]. In food industries, biopolymers with diverse structural and functional properties have been used, including cellulose, starch, chitosan, chitin, guar, locust bean gum, tara gum, glucomannan, xanthan, agar, carrageenan, pectin, alginates, gellan, curdlan, dextran, levan, arabinoxylans, and pullulan [6,7,8]. Nowadays, biodegradable and edible films (BEFs) prepared from different hydrocolloids are gaining popularity worldwide in the food industry to replace synthetic petroleum-based packaging materials [9]. BEFs have been produced from several hydrocolloids, such as chitosan, gelatin, starch, pectin, and other gums [10]. BEFs have been used commercially as wrappings to control the transfer of gases (oxygen and carbon dioxide), moisture and loss of aroma compounds to extend the shelf-life of fresh foods [11]. In addition, BEFs are non-toxic and can help to eliminate food safety concerns and increase the shelf-life of packaged foods [12].

Chitosan (CH) is a linear biopolymer composed of (1,4)-linked-2-amino-deoxy-β-d-glucan units obtained by the deacetylation of chitin, which is the second-most-abundant polysaccharide in nature, after cellulose [13]. CH and its derivatives have excellent film-forming properties to produce BEFs on an industrial scale for food applications [14]. CH-based BEFs have been successfully used as bioactive packaging materials to preserve the quality of different fruits, such as raspberry, apple, kiwi, strawberry, cherimoya, and loquat [12,15,16,17,18,19]. Moreover, CH films have been shown to retard biochemical and microbial quality changes in packaged foods due to their excellent barrier properties against oxygen and water vapor [12]. Apart from food applications, chitosan has been exploited as a natural wound healing dressing, as an antitumor agent, and for safe drug delivery, unaffected by pH changes, to the target sites [20]. Chitosan in the form of core–shell microspheres has been investigated to enhance its cell adhesion and proliferation abilities during wound healing [21]. Antimicrobial peptide (Piscidin-1) incorporated into thermo-responsive chitosan hydrogels could effectively inhibit the growth of *Acinetobacter baumannii* [22]. Gingerol phytosome conjugated with chitosan has demonstrated the sustained release of gingerol from the phytosome under in vitro conditions with antioxidant, anti-inflammatory activities against respiratory infections [23,24]. Therefore, chitosan is an efficient natural material to create complex bioactive substances with several beneficial properties.

Plant extracts rich in phenolic compounds can be employed to enhance the potential bioactivity and barrier properties of BEFs [25]. Chitosan can conjugate with polyphenols to stop oxidative changes and microbial growth in foods. The positively charged amino (-NH_3_) groups of chitosan and the hydroxyl groups of polyphenols interact with negatively charged carboxylic (-COO^−^) groups on the microbial cell membrane to cause a perforation that facilitates the entry of antimicrobial agents into the cytoplasm for the destruction of DNA and RNA of the microbial cell [26]. Cashew leaf (*Anacardium occidentale* L.) extracted in ethanol contains secondary metabolites, such as flavonoids, tannins, saponins, and anthocyanins [27]. Tannin obtained from cashew leaves showed excellent antibacterial and fungicidal properties in an in vitro test [28]. Bioactive compounds from cashew leaves, such as flavonoids and quercetin, have been documented to inhibit pathogenic microorganisms [29]. Cashew leaf extract (CLE) has been reported to inhibit the growth of *Staphylococcus aureus*, *Streptococcus mutans*, *Escherichia coli*, *Candida albican*, and *Aspergillus niger* [28]. Nevertheless, there is no information about the preparation and characterization of bioactive CH films loaded with CLE for food applications. Therefore, the current study was conducted to investigate the desirable antioxidant and antifungal properties under in vitro conditions of CLE obtained via aqueous and ethanolic extraction of immature and mature cashew leaves. The impact of a CLE-fortified CH film on the antifungal, barrier, and optical properties was also examined.

## 2. Materials and Methods

### 2.1. Materials

All chemicals and microbial media used in the experiments were of analytical grade. Dimethyl sulfoxide (DMSO) was purchased from RCI Labscan Limited, Bangkok, Thailand. Chitosan powder (80,000 MW), Tween-80, potato dextrose agar (PDA), and potato dextrose broth (PDB) were obtained from Sigma-Aldrich (St. Louis, MO, USA). The fungal culture, typically *A. niger*, was isolated by the Thailand Bioresource Research Center (TBRC) (Bangkok, Thailand).

### 2.2. Preparation of Cashew Leaf Extract

Cashew leaves (*Anacardium occidentale* L.) were procured from the Ban Bang Klang plantation, Ranong, Thailand, in October 2021. Immature and mature cashew leaves with no apparent damage were collected and transported to the Postharvest Technology and Packaging Laboratory, Mae Fah Luang University, Chiang Rai, Thailand. The cashew leaves were washed with distilled water and dried at 50 °C for 24 h. Immature and mature samples of cashew leaves were powdered and stored in polyethylene zip-lock bags. The powdered immature cashew leaves (ICL) and mature cashew leaves (MCL) were soaked in distilled water and 70% ethanol at a ratio of 1:15 (*w*/*v*). Cashew leaf extract (CLE) samples were extracted into four portions as follows:

Immature aqueous cashew leaves extracted in distilled water (IACLE)
Immature ethanolic cashew leaves extracted in 70% ethanol (IECLE)
Mature aqueous cashew leaves extracted in distilled water (MACLE)
Mature ethanolic cashew leaves extracted in 70% ethanol (MECLE)

The extraction process of the above samples was carried out in a closed chamber of a temperature-controlled orbital shaker (IKA KS 3000 I control, IKA-Werke GmbH & Co., Staufen, Germany) at 150 rpm and 23 °C for 48 h. After the extraction process, all samples were filtered through Whatman No. 4 filter paper (Schleicher & Schuell, Maidstone, England) using a Buchner funnel equipped with a vacuum pump. The filtered CLE samples were concentrated by a rotary evaporator at 40 °C for 20 min. Excess distilled water and 70% ethanol were completely removed from the CLE samples using a nitrogen organomation evaporator (N-EVAP 116, Organomation, MA, USA). Finally, CLE samples were lyophilized using a freeze-dryer (FD 8-55 Chris, Martin Christ Gefriertrocknungsanlagen GmbH, Osterode am Harz, Germany) for 48 h to obtain a dry CLE sample with no moisture or ethanol content. A 5% (*w*/*v*) of each lyophilized CLE sample was prepared in 50% (*w*/*v*) dimethyl sulfoxide and stored at 4 °C prior to in vitro chemical and antifungal analyses [30].

### 2.3. Total Phenolic Content (TPC) and 2,2-Diphenyl-1-picrylhydrazyl Free Radical Scavenging (DPPH) Activity of CLE

The total phenolic content of CLE samples was determined by the Folin–Ciocalteu assay (ISO 14502-1, 2005), and gallic acid was used as the standard. CLE (500 μL) samples were mixed with 2.5 mL of 10% (*w*/*v*) Folin–Ciocalteu reagent and 2 mL of 7.5% (*w*/*v*) sodium carbonate. The mixtures were stirred and incubated in darkness for 1 h at room temperature (25 °C). The absorbance of all the CLE samples was measured at 765 nm using a microplate spectrophotometer (Thermo Fisher scientific, Multiskan GO, USA) [31]. The TPC was expressed as mg gallic acid equivalent (mg GAE/g) of the dry extract [32].

The free radical scavenging activities of CLE samples were analyzed by the 2,2-diphenyl-1-picrylhydrazyl (DPPH) method [33]. A DPPH solution (60 mM) was prepared by dissolving 0.00236 g in 95% ethanol (*v*/*v*). The DPPH solution was mixed with CLE samples (50 μL). Trolox (10,000 μM) was used as a standard solution, and methanol was used as a blank. The mixtures were left at room temperature for 30 min. The absorbance was measured at 517 nm using a microplate spectrophotometer (Thermo Fisher scientific, Multiskan GO, USA) [31]. DPPH activities of CLE samples were expressed as µmol Trolox equivalent (TE)/g of the dry extract [34].

### 2.4. Minimal Inhibitory Concentration (MIC), Minimal Fungicidal Concentration (MFC), and Disk Diffusion Test of CLE Samples

The MIC of all CLE samples was determined by mixing them with PDB (1:1 *v*/*v*) and transferring them into a 96-well microplate. Sample mixtures containing broth and CLE were subjected to serial twofold dilution in the concentration range of 50 to 0.05 µg. A suspension of *Aspergillus niger* spores (10^6^ spores/mL) was loaded into each concentration (1:10 *v*/*v*). The microplate was incubated at 25 °C for 48 h. The MIC was observed from the absorbance at 625 nm against visible fungal growth using a microplate spectrophotometer (Multiskan GO, FisherScientific lda, Porto Salvo, Protugal) [31]. The MFC was determined using the concentration of CLE samples from the MIC that presented an invisible growth of *A. niger*. The concentrations selected from the MIC were sub-cultured on PDA plates and incubated at 25 °C for 48 h. The lowest concentration without any fungal growth was recorded and reported as the MFC of the CLE samples [35]. The disk diffusion test of the CLE samples was analyzed by placing a 6 mm sterile paper disk in the center of the plate inoculated with the *A. niger* suspension (100 µL). Diluted concentrations of CLE samples (10 µL) were placed on the paper disks and incubated at 25 °C. The inhibition zones of the different CLE samples were measured based on mycelium growth [36].

### 2.5. Preparation of a Chitosan Film Supplemented with CLE

Chitosan (CH) films treated with selected CLE treatments, such as MACLE and MECLE samples, revealed potential antioxidant and antifungal properties during in vitro analysis of CLE. CH (2%, *w*/*v*) was prepared in 1% lactic acid and homogenized at 10,000 rpm for 1 h. CLE samples (5%) were prepared in 5 mL dimethyl sulfoxide (50% DMSO). Film-forming solutions (FFS) were prepared by mixing 5 mL of DMSO containing 5% of the CLE sample with 95 mL of CH solution [37]. The FFS were prepared as follows:

CH-CON (2% chitosan without CLE)
CH-MACLE-5 (2% chitosan + 5% CLE from mature leaves extracted in deionized water)
CH-MECLE-5 (2% chitosan + 5% CLE from mature leaves extracted in 70% ethanol)

De-aerated FFS (4 g) were cast onto a rimmed silicone resin plate (50 × 50 mm^2^) and evaporated at room temperature for 24 h prior to being dried in a ventilated oven environmental chamber (model H110K-30DM; Seiwa Riko Co., Tokyo, Japan) at 25.0 ± 0.5 °C and 50 ± 5% relative humidity (RH) for another 24 h [38].

### 2.6. Determination of Antifungal, Barrier, and Optical Properties of Chitosan Films Loaded with CLE

#### 2.6.1. Antifungal Analysis of Chitosan Films without and with CLE

Disk diffusion tests were conducted for analyzing the antifungal activity of the CH film with CLE samples [35]. *A. niger* spores were suspended in sterile distilled water. The fungal strain was activated in nutrient broth for 48 h. The fungal solution of 10^6^/mL spores (100 μL) was mixed with 150 mL of PDA, and then 25 mL of the mixtures was poured into a petri dish. CH films with and without CLE treatments were cut into 8 mm diameter discs and placed in the center of PDA plates incubated at 25 °C for 48 h. Inhibitory activity was observed as the diameter of the clear zones of the PDA plates containing the film samples [36].

#### 2.6.2. Water Vapor Transmission Rate (WVTR), Water Vapor Permeability (WVP), and Gas Transmission Rate (GTR) of Chitosan Films with Added CLE

The films were evaluated for WVTR and WVP using the standard ASTM (1989) test method [12]. The films were placed on stainless WVTR cups comprising silica gel as cover. Then, the films were stuck by an O-ring and adjusted to vacuum with hot paraffin. The sample cups were stored in an environmental chamber at 30 °C and 45% RH. The cups were weighed at 1 h intervals (over 8 h). The WVTR (g/m^2^·h) and the WVP (g·m/m^2^·h·Pa) was calculated using the following equations:

(1) WVTR = (W/T))/A

(2) WVP = (WVTR × L)/ΔP

where W, A, L, and ΔP represent the slope of weight versus time, the area of the film surface (m^2^), the film thickness (m), and the vapor pressure difference between both sides of the film (Pa), respectively.

The oxygen transmission rate (OTR) of the films was measured using an OTR test system (OX2/230, Labthink). Before OTR measurement, the samples were conditioned at a temperature of 21 ± 2 °C and 50 ± 5% RH for 24 h. The OTR of the films was determined according to ASTM D 3985–06 standard at 23 ± 2 °C and 50 ± 5% RH [39]. The OTR was measured after the film was placed in a cell, and oxygen flow was introduced to one side of the film. Oxygen permeability was calculated and represented in units cm^3^/m^2^·day·Pa.

#### 2.6.3. Color Values and Thickness Measurement of Chitosan Films with Added CLE

The color of the CH films with CLE treatment was determined using the Hunter Lab Colorimeter (Miniscan EZ 4500L, Hunter Associates Laboratory, Inc., Reston, VA, USA) and expressed as L* (lightness), a* (redness/greenness), and b* (yellowness/blueness) [19]. The film samples were measured using a hand-held micrometer (Mitutoyo, IL, USA). At least five random areas were selected for each of the film samples, and measurements were taken at five different points. Tests were performed in triplicate, and the average value was calculated in millimeters [40].

#### 2.6.4. Two-Photon Microscopy

Two-photon microscopy was performed to achieve a 3D representation of the internal structure of films with a distribution of the CLE sample through the chitosan polymer matrix. Imaging was carried out with a Plan Apo IR×60 objective (NA: 1.27, Water Immersion, Nikon, Japan) at a scanning speed of 1 frame per second. An IR laser (Chameleon, Coherent) was used to provide a 750 nm excitation. The autofluorescence emission from the CLE was collected on four detection channels (FF01-492/SP-25 (400−492 nm), FF03-525/50-25 (500−550 nm), FF01-575/25-25 (563−588 nm), and FF01-629/56-25 (601−657 nm). Increasing laser intensity was used along with the film depth for 3D images to compensate for thickness. For this method, the films were previously cast onto a coverslip and stored at 100% RH before observation [41].

### 2.7. Statistical Analysis

Analysis of variance (ANOVA) and Duncan’s multiple range test was performed using a statistical program, SPSS (Chicago, IL, USA) v. 10.0. Samples were analyzed at a level of significance of *p* < 0.05 for all the parameters.

## 3. Results and Discussion

### 3.1. Antioxidant and Antifungal Properties of Aqueous and Ethanolic CLE

The total phenolic content (TPC) of 5% CLE solutions of IACLE, IECLE, MACLE, and MECLE samples are presented in Table 1. MECLE samples were higher in TPC than the IACLE, IECLE, and MACLE samples (*p* < 0.05). The TPC of the extracts from mature cashew leaves was in the higher range of 872.2 to 1383.4 (mg GAE/g dry extract) compared to that from immature CLE samples with a lower content of phenolics, regardless of the extraction solvents. It was postulated that the higher TPC obtained in the MECLE sample extracted in 70% ethanol might have migrated inside the cashew leaf cells and washed out along with the cell matrix containing both polar and nonpolar phenolic compounds. The TPC of different extracts from guava leaves, custard apple leaves, and noni leaves showed a higher content of phenolic compounds extracted from mature leaves in aqueous ethanol [42,43,44].

The antioxidant potentials determined by DPPH radicle scavenging activities of 5% CLE solutions of different samples prepared from immature and mature leaves are presented in Table 1. The MECLE sample showed the highest DPPH activity, followed by the MACLE sample and other samples prepared from immature cashew leaves (*p* < 0.05). The higher increment in the DPPH activity of the MECLE sample was in line with the TPC. In addition, immature CLE samples such as IACLE and IECLE ranked the lowest in terms of the DPPH activities, as evidenced by the lower TPC contents (Table 1). Total phenols obtained in guava (leaves and seeds) and pomegranate (peels and seeds) wastes were reported to have the highest 2, 2 diphenyl-1-picrylhydrazyl activities [45].

The antifungal properties of 5% CLE samples analyzed via MIC, MFC, and disk diffusion test are presented in Table 1. MIC and MFC values of the MECLE sample against the growth of *A. niger* were lower than those of the other CLE samples (*p* < 0.05). The lowest MIC (1.5 ± 0.2 μg/100 μL) and MFC (3.25 ± 0.3 μg/100 μL) values attained during the growth inhibition of *A. niger* were attained in MECLE probably due to a higher polyphenolic content of the CLE compared to the other CLE samples. The activities of leaf extracts of cashew (*Anacardium occidentale*) and pawpaw (*Carica papaya*) in vitro were reported to inhibit mycelia growth of *A. niger* and *Aspergillus flavus* [46]. Antimicrobial agents in CLE could inhibit microbial growth by the disruption of DNA and RNA in the cytoplasm [26].

The disk diffusion test of CLE samples that measures the inhibition zone of *A. niger* is shown in Table 1. The inhibition zone of *A. niger* was higher in MECLE samples compared to the other CLE samples (*p* < 0.05). This might be associated with the higher antifungal potential of total phenolic compounds as evidenced by lower MIC and MFC values. The petroleum ether extract from flowers of Hibiscus rosa-sinensis at concentrations of 4 mg/disc and 2 mg/disc displayed the strongest inhibition zones of Staphylococcus aureus [47]. *Avicennia marina* extracts of the roots, fruits, and seeds showed the highest antifungal activity against Aspergillus fumigatus and Candida albicans [48].

### 3.2. Antifungal Potential of CLE-Fortified Chitosan Films

The antifungal activity of chitosan films with added CLE samples is presented in Table 2. The highest inhibition zone was obtained in a chitosan coating combined with 5% MECLE (13.67 ± 1.6 mm), followed by 5% MACLE (11.41 ± 1.61 mm) (*p* < 0.05). A CH-CON film without any CLE treatment showed no inhibition zones of the tested strain of fungi. The MECLE sample revealed a higher in vitro antifungal activity of CLE compared to aqueous extraction (Table 1). The antifungal efficacy of the CH-MECLE-5 film was induced by the addition of CLE that might be embedded with the chitosan matrix to enhance bioactivity. The phenolic compound could inhibit the growth of fungi such as *A. niger*, Penicillium sp., and Fusarium floriferous by changing phenol to a single-carbon atom [49]. Therefore, CH-MACLE-5 was lower in antifungal ability, possibly due to the limited extraction of bioactive compounds in the aqueous medium compared to the 70% ethanolic extraction of CLE. Moreover, tannin, quinones, and flavonoids represented in CLE could disrupt cell membranes and arrest the metabolism of fungi. Chitosan-based active films developed by the incorporation of carvacrol (10 g/L), pomegranate peel extract (PPE, 10 g/L), and carvacrol + PPE (10 g/L of each) showed excellent antifungal properties against different pathogenic microbes [50].

### 3.3. Color Values and Appearance of Chitosan Films Containing CLE

**Figure 1:**
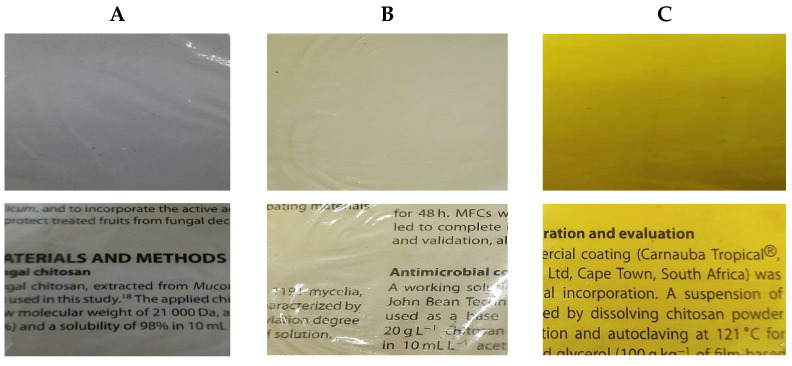
Photographs representing the appearance of different films from chitosan mixed with CLE samples: CH-CON (**A**), CH-MACLE-5 (**B**), and CH-MECLE-5 (**C**). Key: See the footnote of Table 2.

The color of a chitosan film mixed with CLE is expressed as L, a*, and b* values in Table 2. As per the result, CH-CON samples showed the highest lightness (L*) values compared to the films treated with CLE extracted in aqueous and 70% ethanolic solvents from mature cashew leaves (*p* < 0.05). Subsequently, the L* values decreased in CH-MACLE-5 and CH-MECLE-5 film samples due to the addition of CLE. The lowest L* values were attained in the CH-MECLE-5 film due to the higher content of total phenols compared to other chitosan films (*p* < 0.05). The CH-CON sample was more transparent compared to CH-MACLE-5 and CH-MECLE-5 films. In addition, the CH-MECLE-5 film presented higher values of yellow-orange color, as evidenced by +ve a* and +ve b*, than the CH-CON and CH-MACLE-5 films. The chitosan films without CLE display a visually transparent and glossy appearance, while the chitosan film with CLE samples extracted in aqueous and ethanolic solvents showed a yellow-orange appearance (Figure 1). The CH-MECLE-5 film displayed a slightly dark-yellow color, probably due to turbidity caused by the higher phenolic content, compared to the CH-MECLE-5 film. The result was in line with the decreased opacity of chitosan film mixed with natural phenolic extract [51]. A plant polyphenol conjugate with the chitosan matrix affected color because of turbidity in treated films [52]. The incorporation of carvacrol and pomegranate peel extract into chitosan films decreased the transparency due to a higher concentration of total phenols [50].

**Figure 2:**
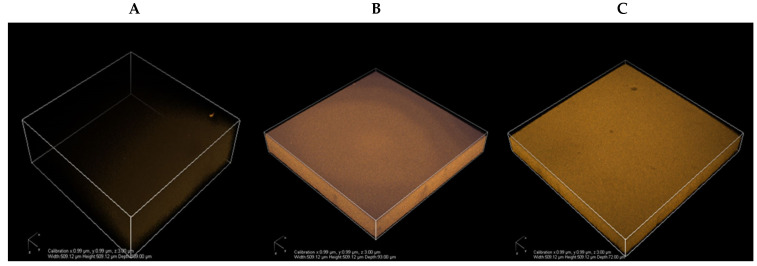
Two-photon 3D micrographs of different films from chitosan mixed with CLE samples: CH-CON (**A**), CH-MACLE-5 (**B**), and CH-MECLE-5 (**C**). Key: See the footnote of Table 2.

Environmental scanning electron microscope (ESME) images of chitosan films treated with CLE samples extracted in aqueous and ethanolic solvents from mature cashew leaves are shown in Figure 2. The distribution of CLE within the chitosan matrix was explored using two-photon microscopy, based on the CLE autofluorescence. The different samples visualized, CH-CON, CH-MACLE-5, and CH-MECLE-5, are shown in Figure 2A–C. ESEM images of the chitosan-CLE films revealed the uniform distribution of CLE on the face of the films in a 3D pattern. The stacking imaging inside the films allowed obtaining a 3D reconstruction of the film structure, with a resolution of about 0.1 μm (Figure 2). These 3D views of the inner structure of the chitosan films show that, without wavelength selection, the main autofluorescence comes from CLE. A CH-CON film without CLE also displayed autofluorescence to some degree. CH-MECLE-5 had noticeably higher fluorescence compared to the CH-MACLE sample and CH-CON films. Additionally, the CH-MACLE-5 film displayed lighter fluorescence, possibly due to the lower amount of CLE polyphenols. Moreover, all the films, such as chitosan and chitosan-treated CLE films, showed a uniform distribution but variation in the intensity of fluorescence, indicating that all of the CLE was uniformly and finely dispersed in the matrix. ESEM images of chitosan treated with lignin depicted the variation in the fluorescence intensity due to the uneven distribution of lignin in the composite films [42].

### 3.4. Impact of CLE-Enriched Chitosan Films on the Thickness, Water Vapor Transmission Rate (WVTR), the Water Vapor Permeability (WVP), and the Oxygen Transmission Rate (OTR)

The thickness, WVTR, and WVP properties of chitosan films supplemented with MACLE and MECLE samples are presented in Table 3. CH-MECLE-5 had the highest value of film thickness compared to CH-MACLE-5 and CH-CON films (*p* < 0.05). The CH-CON film without the addition of CLE measured the lowest thickness compared to the treated films (*p* < 0.05). Chitosan blended with *Pistacia terebinthus* extract containing polyphenols could increase the mechanical and barrier properties of the resultant film [37]. The WVTR, WVP, and OTR were the lowest in the CH-MECLE-5 film compared to other samples (*p* < 0.05) (Table 3).

A chitosan film combined with carvacrol peel extracts could reduce the WVP by the adjustment of the hydrophobic fraction [50]. The WVP, WVTR, and OTR of the chitosan film blended with plant extracts were due to phenolic compounds, which can decrease the hydrophilicity of a chitosan film [53]. A chitosan film incorporated with the plant extract of *Lycium barbarum* showed lower WVP and OTR than the control film because the hydrophobicity of the bioactive compound fits into the chitosan matrix, thereby delaying the transmission of water molecules through the film [54]. Additionally, the WVTR was significantly decreased in chitosan films containing essential oils (EOs) or other plant extracts or to which carvacrol (0.5, 1.0, and 1.5% *v*/*v*) was added [55]. Several reports have shown a decreased WVTR using EOs and plant extracts such as tea tree essential oil, carvacrol, cinnamon essential oil, and turmeric EO in chitosan coating films, possibly due to the hydrophobicity of the EO particles and their ability to occupy the amorphous regions of the films [56,57,58,59].

## 4. Conclusions

Immature and mature cashew leaves were extracted in aqueous and 70% ethanolic solvents. Higher TPC and DPPH values were attained in the 5% MECLE sample. The in vitro MIC, MFC, and zone of inhibition against *A. niger* were marked the highest in the 5% MECLE sample. CH-CON showed the highest L* and the lowest a* and b* values. However, the lightness of the CH film decreased with the addition of the 5% MECLE sample. Higher film thickness was measured in the CH-MECLE-5 film than in the CH-CON film. The CH-MECLE-5 film exhibited excellent antifungal activity. Film appearance visualized under two-photon microscopy displayed uniform fluorescence of CLE dispersed in the CH-MECLE-5 film matrix. Lower WVTR, WVP, and OTR were recorded in the CH-MECLE-5 film compared to the CH-CON film. Therefore, a chitosan film blended with a 5% MECLE sample could form a bioactive film with excellent antifungal, moisture, and gas barrier properties. Thus, the chitosan film loaded with CLE should be further investigated for food packaging subject to the assessment of legal standards to claim it as an eco-friendly substitute to plastic films in the quality preservation of fresh fruits during postharvest storage.

## Figures and Tables

**Table 1:** Total phenols and antioxidant activity of different CLE samples and their effect on the radial growth and inhibition zone of *A. niger*.

CLE Samples	TPC (mg GAE/g)	DPPH	Inhibition Zone of CLE (mm)	MIC (μg/100 μL)	MFC (μg/100 μL)
IACLE	644.6 ± 10.5 ^d^	381.7 ± 2.4 ^d^	9.2 ± 0.2 ^d^	25.0 ± 0.3 ^a^	50.0 ± 0.4 ^a^
IECLE	795.2 ± 16.9 ^c^	392.4 ± 3.7 ^c^	10.1 ± 0.2 ^c^	6.25 ± 0.2 ^b^	12.5 ± 0.6 ^b^
MACLE	872.2 ± 17.2 ^b^	401.3 ± 1.1 ^b^	12.3 ± 0.2 ^b^	3.12 ± 0.1 ^c^	6.25 ± 0.2 ^c^
MECLE	1383.4 ± 42.1 ^a^	781.2±2.1 ^a^	14.2 ± 0.4 ^a^	1.5 ± 0.2 ^d^	3.25 ± 0.3 ^d^

Values are the mean ± the standard deviation (*n* = 6). Different superscripts within the same column followed by different letters (^a–d^) indicate a significant difference (*p* < 0.05). IACLE, IECLE, MACLE, and MECLE presented immature and mature cashew leaves extracted in aqueous and 70% ethanolic solvents.

**Table 2:** Color values and inhibition zones of *A. niger* by chitosan films supplemented with CLE.

Film Samples	L*	a*	b*	Inhibition Zone (mm)
CH-CON	85.9 ± 1.5 ^a^	1.6 ± 0.3 ^c^	−0.5 ± 0.3 ^c^	-
CH-MACLE-5	80.4 ± 1.1 ^b^	2.4 ± 0.1 ^b^	15.0 ± 0.6 ^b^	10.4 ± 1.6 ^b^
CH-MECLE-5	72.5 ± 0.5 ^c^	6.2 ± 0.2 ^a^	57.0 ± 0.8 ^a^	13.7 ± 1.3 ^a^

Values are the mean ± the standard deviation (*n* = 6). Different superscripts within the same column followed by different letters (^a–c^) indicate a significant difference (*p* < 0.05). CH-CON (2% chitosan, without any treatment), CH-MACLE-5 (2% chitosan + 5% aqueous CLE), and CH-MECLE-5 (2% chitosan + 5% CLE extracted in 70% ethanol).

**Table 3:** Thickness, moisture barrier, and oxygen barrier properties of chitosan films incorporated with CLE.

Films	Thickness (mm)	WVTR (g/m^2^·h)	WVP (g·m/m^2^·h·Pa)	OTR (cm^3^·/m^2^·day·Pa)
CH-CON	0.0436 ± 0.001 ^c^	3.1 ± 0.5 ^a^	8.5 ± 0.3 × 10^−8 a^	1.28 × 10^8^ ± 5.8 × 10^6 a^
CH-IECLE-5	0.0579 ± 0.002 ^b^	1.9 ± 0.3 ^b^	6.9 ± 0.5 × 10^−8 ab^	1030 ± 247.48 ^b^
CH-MECLE-5	0.0604 ± 0.001 ^a^	1.3 ± 0.2 ^c^	5.1 ± 0.8 × 10^−8 c^	155 ± 24.04 ^c^

Values are the mean ± the standard deviation (*n* = 6). Different superscripts within the same column followed by different letters (^a–c^) indicate a significant difference (*p* < 0.05). CH-CON (2% chitosan, without any treatment), CH-MACLE-5 (2% chitosan + 5% aqueous CLE), and CH-MECLE-5 (2% chitosan + 5% CLE extracted in 70% ethanol). WVTR, water vapor transmission rate; WVP, water vapor permeability; OTR, oxygen transmission rate.

## Data Availability

The data presented in this study are available on request from the corresponding author.
